# Combined Administration of Nitric Oxide and Hydrogen into Extracorporeal Circuit of Cardiopulmonary Bypass as a Method of Organ Protection during Cardiac Surgery

**DOI:** 10.17691/stm2023.15.5.02

**Published:** 2023-10-30

**Authors:** V.V. Pichugin, A.V. Derugina, S.E. Domnin, A.S. Shirshin, S.A. Fedorov, S.N. Buranov, S.A. Jourko, M.V. Ryazanov, D.A. Danilova, Yu.D. Brichkin

**Affiliations:** MD, DSc, Professor, Anesthesiologist-Resuscitator; Specialized Cardiosurgical Clinical Hospital named after Academician B.A. Korolev, 209 Vaneeva St., Nizhny Novgorod, 603136, Russia; Professor, Department of Anesthesiology, Intensive Care Medicine, and Transfusiology; Privolzhsky Research Medical University, 10/1 Minin and Pozharsky Square, Nizhny Novgorod, 603005, Russia; DSc, Associate Professor, Head of the Department of Physiology and Anatomy; National Research Lobachevsky State University of Nizhny Novgorod, 23 Prospect Gagarina, Nizhny Novgorod, 603022, Russia; MD, PhD, Anesthesiologist-Resuscitator; Specialized Cardiosurgical Clinical Hospital named after Academician B.A. Korolev, 209 Vaneeva St., Nizhny Novgorod, 603136, Russia; PhD, Leading Researcher; Russian Federal Nuclear Center — All-Russian Scientific Research Institute of Experimental Physics (RFNC-VNIIEF), 37 Prospect Mira, Sarov, 607188, Russia; MD, PhD, Cardiovascular Surgeon; Specialized Cardiosurgical Clinical Hospital named after Academician B.A. Korolev, 209 Vaneeva St., Nizhny Novgorod, 603136, Russia; PhD, Leading Researcher; Russian Federal Nuclear Center — All-Russian Scientific Research Institute of Experimental Physics (RFNC-VNIIEF), 37 Prospect Mira, Sarov, 607188, Russia; MD, PhD, Head of the Unit, Cardiovascular Surgeon; Specialized Cardiosurgical Clinical Hospital named after Academician B.A. Korolev, 209 Vaneeva St., Nizhny Novgorod, 603136, Russia; MD, PhD, Head of the Unit, Cardiovascular Surgeon; Specialized Cardiosurgical Clinical Hospital named after Academician B.A. Korolev, 209 Vaneeva St., Nizhny Novgorod, 603136, Russia; Associate Professor, Department of Hospital Surgery named after B.A. Korolev; Privolzhsky Research Medical University, 10/1 Minin and Pozharsky Square, Nizhny Novgorod, 603005, Russia; Senior Tutor, Department of Physiology and Anatomy; National Research Lobachevsky State University of Nizhny Novgorod, 23 Prospect Gagarina, Nizhny Novgorod, 603022, Russia; MD, DSc, Consulting Professor; Specialized Cardiosurgical Clinical Hospital named after Academician B.A. Korolev, 209 Vaneeva St., Nizhny Novgorod, 603136, Russia

**Keywords:** nitric oxide, hydrogen, cardiopulmonary bypass, heart surgery

## Abstract

**The aim** is to study the effect of combined introduction of nitric oxide and hydrogen into the extracorporeal circuit of cardiopulmonary bypass (CPB) for antioxidant activity and organ damage during cardiac surgery.

**Materials and Methods:**

The study included 91 patients who underwent heart surgery under CPB. The patients were randomized into 3 groups: group 1 comprised 30 patients (control); group 2 consisted of 33 patients with an isolated supply of nitric oxide (40 ppm) to the extracorporeal circuit; group 3 included 28 patients with a combined supply of nitric oxide (40 ppm) and hydrogen (1.2 ppm) into the extracorporeal circuit. The intensity of lipid peroxidation processes was studied by the content of diene conjugates (DC), triene conjugates (TC), Schiff bases (SB) in blood plasma; erythrocyte aggregation was also examined. The studies were carried out at the following stages: stage 1 (initial) — after induction of anesthesia; stage 2 — before CPB; stage 3 — 5 min after CPB initiation; stage 4 — at the 30^th^ minute of CPB; stage 5 — at the 60^th^ minute of CPB; stage 6 — at the 90^th^ minute of CPB; stage 7 — at CPB termination; stage 8 — at the end of the operation.

**Results:**

The content of DC increased statistically significantly at the 90^th^ minute of CPB to 1.093±0.573 rel. units (M±SD) in patients of group 1; to 0.322±0.047 rel. units in group 2; to 0.287±0.003 rel. units in group 3, while the DC content was statistically significantly lower in patients of groups 2 and 3 compared to group 1. A statistically significant increase in the content of TC compared to the initial value was observed at the 90^th^ minute of CPB in group 1 (up to 0.334±0.114 rel. units), while the content of TC was statistically significantly lower in patients of groups 2 and 3. A statistically significant growth in the content of SB occurred at the 90^th^ minute of CPB in patients of group 1 up to 33.324±15.640 rel. units. This indicator was statistically significantly lower in groups 2 and 3 relative to the patients of group 1. The dynamics of erythrocyte aggregation in patients of group 1 showed statistically significant growth of this indicator from the start of CPB to the end of the operation (from 44.8±1.4 to 73.1±2.2%). The statistically significant difference from the indicator at the beginning of the operation started at the 30^th^ minute of CPB and lasted until the end of the operation. In patients of group 2, it decreased statistically significantly during CPB (from 56.5±2.3% before the CPB initiation to 47.4±1.2% at the CPB termination); in patients of group 3, it was decreasing from the 60^th^ minute of CPB to the end of the operation and was statistically significantly lower than in patients of both groups 1 and 2. No postoperative complications were noted (acute heart failure, acute respiratory failure, multiple organ failure) in patients of groups 2 and 3. A statistically significant decrease in both the duration of mechanical ventilation and stay in the intensive care unit was registered in group 3 compared to group 2.

**Conclusion:**

The combined use of gaseous nitric oxide and hydrogen during CPB allowed a statistically significant decrease in the level of activation of lipid peroxidation and erythrocyte aggregation, which ensured a higher level of organ protection during cardiac surgery, faster activation of patients, and a shorter stay in the intensive care unit.

## Introduction

The use of cardiopulmonary bypass (CPB) is associated with the activation of lipid peroxidation (LPO) of biomembranes where oxygen free radicals play an inducing role. The LPO products are capable of damaging the cell energy apparatus, dissociating the respiration-coupled oxidative phosphorylation, changing the activity of the membrane-linked enzymes, increasing the permeability of cellular membranes, impairing the systems of cell regulation and division, i.e. can cause systemic damage to a cell [1-3].

It is known that there are two ways of oxygen utilization in a cell — by oxidase and oxygenase enzymes. In the first case, oxygen is reduced to the water molecule on mitochondrial respiratory chains; in the second case, it forms reactive oxygen species (ROS) which are extremely active, capable of damaging cellular structures either directly or indirectly through activation of the LPO processes. Any extreme impact (for example, surgical intervention) causes the organism to respond first by the increased intensity of metabolism facilitating the adaptation to new conditions. If the load exceeds protection capability, failure occurs with the development of the oxidative stress. Failure of the organism to neutralize the generated ROS results in the oxidative damage to organs and tissues [4, 5].

Extensive surgical intervention, impairing normal oxygen consumption and energy generation, triggers a cascade of pathophysiological reactions, the basis of which is an alteration of the free-radical oxidation processes, i.e. the dynamic balance is shifted towards the stimulation of ROS generation and accumulation of underoxidized toxic products intensifying the damaging effect [6].

Intensification of free radical processes plays an important role in the pathogenesis of intraorgan injuries in the operations under CPB. In the course of surgical intervention, tissues are subject to hypoxia and reoxygenation, which causes activation of the LPO processes and changes in the antioxidative properties of blood [7]. Oxidative injuries of membranes caused by the enhanced ROS generation occupy an important place among ischemic and reperfusion injuries, influence essentially the outcome of the operation, and may occur immediately both after CPB and in the early postoperative period [8].

Nitric oxide possesses antioxidant effect by inhibiting oxidative reactions, increasing the activity of antioxidant enzymes and expression of the genes encoding them. Nitric oxide can slow down lipid peroxidation acting as a scavenger of the oxygen radicals. Therefore, the interaction between superoxide anion and nitric oxide may be a biologically important way to detoxification of potentially dangerous reactive oxygen species [9]. Besides, molecular hydrogen is known to bind free radicals of hydroxyl and peroxynitrite. The action of these radicals on the critically important biomolecules of nucleic acids, lipoproteins of the cell membranes, and cellular organelles leads to the damage of myocardium during operations with CPB [10]. Presently, there are no clinical investigations devoted to the combined application of nitric oxide and hydrogen during the operations with CPB, which determines the significance of the research.

**The aim** is to study the effect of combined introduction of nitric oxide and hydrogen into the cardiopulmonary bypass circuit for antioxidant activity and organ damage during cardiac surgery.

## Materials and Methods

The course of intra- and postoperative period has been analyzed in 91 patients, who undergone operations on the heart under CPB (operations on the heart valves, aortocoronary bypass + correction of valvular pathology). The study was approved by the Ethical Committee of the Specialized Cardiosurgical Clinical Hospital named after Academician B.A. Korolev (Nizhny Novgorod, Russia) and complies with the Declaration of Helsinki (2013). Informed written consent was obtained from each patient.

The patients were randomly divided into 3 groups: group 1 comprised 30 patients (control); group 2 consisted of 33 patients with an isolated supply of nitric oxide (40 ppm) to the extracorporeal circuit during CPB; group 3 included 28 patients with a combined supply of nitric oxide (40 ppm) and hydrogen (1.2 ppm) into the extracorporeal circuit during CPB. The description of patients is presented in Table 1. No statistically significant differences between the groups by gender, age, and severity of the state have been found (p>0.05).

**T a b l e 1.** Clinical description of the examined patients

Indicator	Group 1 (n=30)	Group 2 (n=33)	Group 3 (n=28)
Sex (n/%):			
male	12/40.0	16/48.5	17/60.7
Female	18/60.0	17/51.5	11/39.3
Age (years), M±SD	54.1±1.4	59.5±1.4	58.5±1.1
Functional class (NYHA) (n/%):			
III	28/93.3	26/78.8	21/75.0
IV	2/6.6	7/21.1	7/25.0
Left ventricular ejection fraction (%), M±SD	56.0±1.5	54.4±1.4	50.4±1.6

All patients have been operated on the heart valves or undergone combined operations on the valves and coronary arteries. Normothermic CPB was used in all operations, and in order to protect the myocardium, a combined crystalloid pharmaco-cold cardioplegia using Custadiol solution (Germany) was applied. The duration of CPB (M±SD) was 100.30±30.25 min in group 1; 124.60±39.90 min in group 2; 117.40±45.05 min in group 3; aortic cross-clamp time was equal to 75.6±23.1, 93.1±31.9, and 89.3±31.8 min, respectively. Statistically significant differences between the groups have not been found.

In patients of group 1 (control), CPB was carried out according to the protocol adopted at the Specialized Cardiosurgical Clinical Hospital named after Academician B.A. Korolev. Gaseous nitric oxide (40 ppm) for patients of group 2 was supplied to the line delivering gas to the oxygenator of the extracorporeal circulation system (ECS). The Tianox apparatus (RFNC VNIIEF, Russia) was used as a generator of gaseous nitric oxide; nitric oxide dosing was implemented by means of an in-line monitor of the generating device. Nitric oxide delivery to the CPB circuit was started when the rated perfusion capacity was reached and continued to CPB termination. Patients of group 3 were supplied with a combination of gaseous nitric oxide (40 ppm) and molecular hydrogen (1.2 ppm) to the line of delivering gases to the ECS oxygenator. The Tianox apparatus was used as a generator of gaseous nitric oxide, hydrogen was generated using the BozonH2/O3 UV control apparatus (Econica Medical Engineering, Ukraine). The in-line monitors of the generating systems were used for dosing the gases. The connection diagram of gas supply to the extracorporeal circuit is presented in the Figure.

**Figure:**
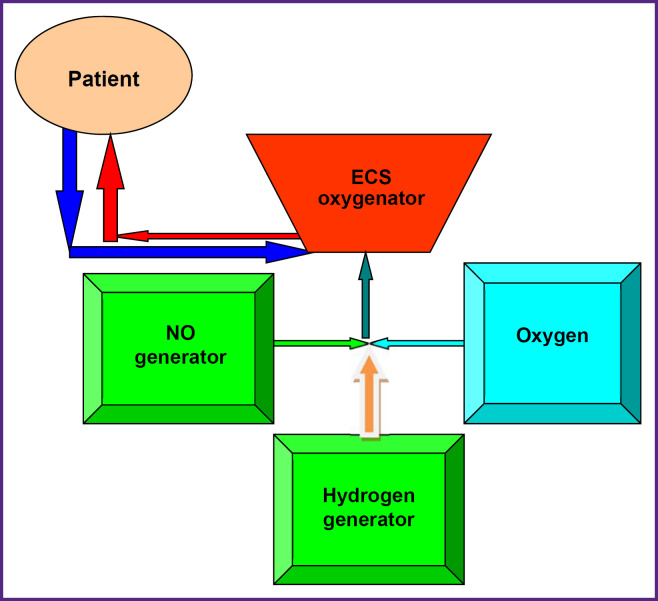
The schematic presentation of the combined connection of nitric oxide and molecular hydrogen generators ECS — extracorporeal circulation system

Once the rated perfusion flow was reached, 40 ppm of nitric oxide and 1.2 ppm of hydrogen were supplied to the extracorporeal circuit. This protocol of nitric oxide and hydrogen supply was maintained throughout the CPB application. Gas delivery to the extracorporeal circulation was stopped after the recovery of effective cardiac activity 5 min before CPB termination.

The intensity of LPO processes was studied by the content of diene conjugates (DC), triene conjugates (TC), Schiff bases (SB) in blood plasma; erythrocyte aggregation was also examined. The studies were carried out at the following stages: stage 1 (initial) — after induction of anesthesia; stage 2 — before CPB; stage 3 — 5 min after CPB initiation; stage 4 — at the 30^th^ minute of CPB; stage 5 — at the 60^th^ minute of CPB; stage 6 — at the 90^th^ minute of CPB; stage 7 — at CPB termination; stage 8 — at the end of the operation. The content of DC, TC, and SB in plasma was determined using I.A. Volchegorsky’s method (1989). Heptane-isopropanol mixture (1:1) was added to blood plasma, shaken, and supplemented with 1 ml of an aqueous HCL solution (pH_2_) and 2 ml of heptane. After the resulting mixture was settled and stratified into phases, optical densities (E) were measured on SF-2000 spectrophotometer (OKB Spectr, Russia), evaluating each phase at the following wavelengths: 220 nm (absorption of isolated double bonds), 232 nm (DC absorption), 278 nm (TC absorption), 400 nm (SB absorption). In the process of the study, the preservation of erythrocytes in patients of groups 2 and 3 was determined by the aggregation properties of erythrocytes. Erythrocyte aggregation was examined using optical microscopy technique by counting single erythrocytes and their aggregates [11]. The solution of dextran blue T-2000 in the dose of 20 mg/ml (GE HealthCare, USA) in Tris-HCl buffer (pH 7.4) was used as a stimulator of aggregation. The washed erythrocytes were diluted with the dexran solution (1:10 by volume) and the number of non-aggregated erythrocytes was counted in the Goryaev chamber. A total number of erythrocytes in a sample was calculated in isotonic NaCl solution. The level of aggregation (A) was estimated by the formula:

A=100%−(the number of free (non-aggregated) erythrocytes  × total number of erythrocytes−1×100%).

Additionally, the course of the postoperative period was studied as a clinical indicator.

**Statistical analysis** was performed using Microsoft Excel 2003 and Statistica 6.0 software. Calculations of the Fisher’s exact criterion for fourfold tables were done on-line on the https://medstatistic.ru/ site. The character of data distribution was assessed using Kolmogorov- Smirnov and Shapiro-Wilk tests since these tests give mainly similar but sometimes different statistics. For the statistical analysis of the data following the law of normal distribution, we used the ANOVA analysis and Dunnett’s test taking into consideration the effect of multiple comparisons with the control group [12]. The results of all tests were considered statistically significant at p<0.05.

## Results

The dynamics of the DC level during the operation is presented in Table 2. Patients of group 1 did not show any statistically significant changes of this indicator before CPB, but its statistically significant rise was observed at the 90^th^ minute of CPB (1.093±0.573 rel. units) with a further decrease by the end of the operation (0.336±0.041 rel. units). The dynamics of the DC level in group 2 also reflected absence of statistically significant changes at all stages of the operation relative to the initial value. Notably that the DC level in group 2 at the 90^th^ minute of CPB was 3 times lower than in patients of group 1. The dynamics of the DC level in patients of group 3 demonstrated absence of statistically significant changes before CPB and also its smooth decrease before perfusion (0.257±0.043 rel. units) and at the end of the operation (0.208±0.050 rel. units). The content of DC at the 90^th^ minute of CPB was statistically significantly lower in patients of group 3 than in group 1.

**T a b l e 2.** Dynamics of diene conjugate content in the examined patients (rel. units), M±SD

Operation stage	Group 1 (control)	Group 2 (NO supply)	Group 3 (NO + H_2_ supply)
Beginning of the operation	0.299±0.059	0.289±0.015	0.278±0.033
Before CPB initiation	0.336±0.050	0.221±0.039	0.257±0.043
5 min of CPB	0.291±0.025	0.268±0.017	0.233±0.036
30 min of CPB	0.523±0.189	0.270±0.022	0.601±0.436
60 min of CPB	0.370±0.073	0.284±0.017	0.340±0.025
90 min of CPB	1.093±0.573**	0.322±0.047*	0.287±0.003*
CPB termination	0.379±0.054	0.308±0.025	0.268±0.046
The end of the operation	0.336±0.041	0.304±0.035	0.208±0.050

* statistically significant difference from the control group; ** statistically significant difference from the indicator at the beginning of the operation.

The dynamics of the TC level during the operation is presented in Table 3. In patients of group 1, there were no statistically significant changes of this indicator before CPB, but its statistically significant growth (from 091 ±0.013 to 0.334±0.114 rel. units) was observed at the 90^th^ minute of CPB, with a further decrease by the end of the operation (up to 0.143±0.092 rel. units). In patients of group 2, the dynamics of the TC level reflected the absence of statistically significant changes of this parameter during the operation: its concentration prior to CPB was 0.128±0.056 rel. units; then, it raised to 0.131±0.044 rel. units by the 90^th^ minute of CPB and returned to the initial values at the end of the operation (0.080±0.015 rel. units). It should be noted that the TC level was statistically significantly lower at the 90^th^ minute of CPB in patients of group 2 than in group 1. The TC level at the 30^th^ minute in patients of group 3 was statistically significantly higher than in group 1, but by the 90^th^ minute of CPB there was a statistically significant decrease in this indicator (0.110±0.020 rel. units) in comparison with group 1.

**T a b l e 3.** Dynamics of triene conjugate contents in the examined patients (rel. units), M±SD

Operation stage	Group 1 (control)	Group 2 (NO supply)	Group 3 (NO + H_2_ supply)
Beginning of the operation	0.106±0.018	0.082±0.012	0.132±0.023
Before CPB initiation	0.091±0.013	0.128±0.056	0.102±0.007
5 min of CPB	0.087±0.019	0.087±0.017	0.083±0.018
30 min of CPB	0.126±0.065	0.098±0.021	0.294±0.204*
60 min of CPB	0.148±0.049	0.083±0.016	0.134±0.050
90 min of CPB	0.334±0.114**	0.131±0.044*	0.110±0.020*
CPB termination	0.082±0.017	0.088±0.018	0.112±0.006
The end of the operation	0.143±0.092	0.080±0.015	0.131±0.015

* statistically significant difference from the control group; ** statistically significant difference from the indicator at the beginning of the operation.

The dynamics of the SB level during the operation is presented in Table 4. In patients of group 1, there were no statistically significant changes of this indicator before CPB and its statistically significant growth (from 15.190±4.715 to 33.324±15.640 rel. units) was noted at the 90^th^ minute of CPB with a further decrease by the end of the operation to the value of 8.204±1.948 rel. units. The dynamics of the SB level in patients of group 2 was characterized by the absence of statistically significant changes of this indicator throughout the operation. At the same time, the SB level was statistically significantly lower (11.483±4.688 rel. units) at the 90^th^ minute of CPB relative to the patients of group 1. The SB level in group 3 was statistically significantly lower than that in group 1 at the 60^th^, 90^th^ minutes, and further up to the end of the operation.

**T a b l e 4.** Dynamics of Schiff bases content in the examined patients (rel. units), M±SD

Operation stage	Group 1 (control)	Group 2 (NO supply)	Group 3 (NO + H_2_ supply)
Beginning of the operation	11.997±3.858	14.872±3.841	4.893±0.921
Before CPB initiation	15.190±4.715	15.385±3.495	31.900±30.232
5 min of CPB	10.601±1.449	15.072±4.451	8.512±1.613
30 min of CPB	12.518±3.407	18.309±5.205	19.768±13.567
60 min of CPB	20.319±6.703	10.803±2.367	6.296±0.022*
90 min of CPB	33.324±15.640**	11.483±4.688*	4.331±1.235*
CPB termination	12.698±2.468	14.423±2.305	4.135±0.230*
The end of the operation	8.204±1.948	10.561±2.579	3.348±1.627*

* statistically significant difference from the control group; ** statistically significant difference from the indicator at the beginning of the operation.

Changes of erythrocyte aggregation during the operation are presented in Table 5. In patients of group 1, there were no statistically significant changes in this parameter before CPB, but beginning with the 30^th^ minute of CPB and up to the end of the operation, this indicator was observed to grow from 44.8±1.4 to 73.1 ±2.2%. Erythrocyte aggregation in patients of group 2 decreased during CPB from 56.5±2.3% before the beginning to 47.4±1.2% at its termination. Erythrocyte aggregation in patients of group 3 was decreasing throughout the operation and was statistically significantly lower than in patients of groups 1 and 2 beginning from the 30^th^ minute of CPB and to the end of the operation. It should be noted that at the beginning of the operation, erythrocyte aggregation in group 3 was statistically significantly higher than in group 1. A statistically significant decrease in erythrocyte aggregation in patients of group 3 provided better microcirculation and oxygen supply to the organs and tissues in the process of CPB and at its termination.

**T a b l e 5.** Dynamics of erythrocyte aggregation during the operation (%), M±SD

Operation stage	Group 1 (control)	Group 2 (NO supply)	Group 3 (NO + H_2_ supply)
Beginning of the operation	37.3±2.5	62.7±2.9	73.1±2.6*
Before CPB initiation	44.8±1.4	56.5±2.3	70.7±2.9*
5 min of CPB	51.0±1.2	55.4±3.0	43.4±1.3
30 min of CPB	61.7±1.5**	49.9±2.2	40.8±2.7*
60 min of CPB	63.9±1.3**	50.7±1.3	30.7±2.7*
90 min of CPB	65.1±2.8**	49.5±1.4	24.7±3.0*
CPB termination	70.0±1.6**	47.4±1.2	20.4±1.2*
The end of the operation	73.1±2.2**	42.8±3.1	18.7±1.5*

* statistically significant difference from the control group; ** statistically significant difference from the indicator at the beginning of the operation.

Clinical outcomes are presented in Table 6. Acute cardiac failure rate in patients of groups 2 and 3 was statistically significantly (Fisher’s exact test) lower (p=0.046) relative to group 1. Statistically significant differences in the acute respiratory and multiple organ failures rate have not been detected between the groups 2 and 3 compared to group 1. Besides, no complications in the early postoperative period were observed in patients of group 2 and 3.

**T a b l e 6.** Clinical outcomes of the examined patients

Characteristics	Group 1 (control)	Group 2 (NO supply)	Group 3 (NO + H_2_ supply)
Duration of ALV after the operation (h), M±SD	8.0±0.5	6.9±0.7	4.7±0.5**
Acute heart failure (n/%)	4/13.3	0/0*	0/0*
Acute respiratory failure (n/%)	3/10.0	0/0	0/0
Multiple organ failure (n/%)	2/6.7	0/0	0/0
Length of intensive care unit stay (h), M±SD	40.0±2.3	33.2±1.1	27.1±1.5**
Mortality (%)	0/0	0/0	0/0
Length of hospital stay (days), M±SD	11.0±2.7	10.1±2.1	9.5±2.0

* statistically significant difference with group 1; ** statistically significant difference between group 2 and 3.

As follows from the data given in Table 6, patients of group 3 were noted to have statistically significantly shorter duration of artificial lung ventilation after the operation and, therefore, a shorter stay in the intensive care unit (ICU) in comparison with group 2.

Thus, a combined application of gaseous nitric oxide and hydrogen during CPB allowed us to statistically significantly reduce the level of LPO activation and erythrocyte aggregation during the operation and CPB, which provided a higher level of organ protection in cardiac-surgical intervention, faster patient activation, and a shorter ICU stay.

## Discussion

The effect of the isolated application of nitric oxide and its combination with hydrogen has been evaluated in comparison with the control group, which was supported in compliance with standard protocols not including these agents.

The effective use of isolated introduction of gaseous nitric oxide to the CPB circuit has been noted in a number of publications in recent years [13-15]. However, the emphasis was made there on the cardioprotective effect of the preparation during cardiac-surgical intervention, but the influence of nitric oxide on the LPO processes and erythrocyte condition has not been investigated, although a systemic damaging effect of the LPO products on a cell is well known. The data obtained by us in the process of the study demonstrate the reduction of the LPO indicators during CPB caused by the isolated use of gaseous nitric oxide. Nitric oxide also significantly influenced the condition of erythrocytes reducing their aggregation in the course of the operation under CPB.

Previous clinical studies devoted to the isolated application of molecular hydrogen in cardiac surgery are limited to its inhalation application at the stages before or after CPB. The analysis of the results of venous and arterial blood tests has shown that the decrease in LPO product concentration in the postoperative period in the group of patients receiving hydrogen inhalations was associated with a direct antioxidative action of the molecular hydrogen and its possible indirect impact by modulating the concentration of reactive oxygen species being natural signaling messengers important for the regulation of cell activity [10]. Besides, the application of molecular hydrogen has been shown to maintain the ATP concentration and to increase statistically significantly the 2,3-DPG content in arterial blood erythrocytes relative to the control group [16]. Investigations concerning the supply of molecular hydrogen to the CPB circuit, the assessment of its effect on the LPO processes, and the erythrocyte condition have not been performed so far.

The data published by us are a pilot study devoted to a combined application of two gases, nitric oxide and molecular hydrogen delivered simultaneously to the CPB machine circuit during cardiac-surgical interventions. The combined application of these agents has been shown to influence effectively not only the decrease in the LPO processes and the erythrocyte condition but clinical outcomes of the operation as well. Based on the results of this study, a patent application for the invention No.2022130057 “The method of preventing organ damage while conducting intraoperative cardiopulmonary bypass” of November 18, 2022 was filed.

## Conclusion

The conducted study has shown that the combined use of gaseous nitric oxide and hydrogen during CPB allowed us to statistically significantly decrease the level of activation of lipid peroxidation and erythrocyte aggregation during the operation, which ensured a higher level of organ protection during cardiac surgery, faster activation of patients, and a shorter stay in the ICU.
